# Disentangling the roles of aneuploidy, chromosomal instability and tumour heterogeneity in developing resistance to cancer therapies

**DOI:** 10.1007/s10577-023-09737-5

**Published:** 2023-09-18

**Authors:** Joana Reis Andrade, Annie Dinky Gallagher, Jovanna Maharaj, Sarah Elizabeth McClelland

**Affiliations:** Barts Cancer Institute, Queen Mary University of London, Charterhouse Square, London, EC1M6BQ England

**Keywords:** Aneuploidy, chromosomal instability, intratumour heterogeneity, therapy resistance, tumour evolution, cancer

## Abstract

Aneuploidy is defined as the cellular state of having a number of chromosomes that deviates from a multiple of the normal haploid chromosome number of a given organism. Aneuploidy can be present in a static state: Down syndrome individuals stably maintain an extra copy of chromosome 21 in their cells. In cancer cells, however, aneuploidy is usually present in combination with chromosomal instability (CIN) which leads to a continual generation of new chromosomal alterations and the development of intratumour heterogeneity (ITH). The prevalence of cells with specific chromosomal alterations is further shaped by evolutionary selection, for example, during the administration of cancer therapies. Aneuploidy, CIN and ITH have each been individually associated with poor prognosis in cancer, and a wealth of evidence suggests they contribute, either alone or in combination, to cancer therapy resistance by providing a reservoir of potential resistant states, or the ability to rapidly evolve resistance. A full understanding of the contribution and interplay between aneuploidy, CIN and ITH is required to tackle therapy resistance in cancer patients. However, these characteristics often co-occur and are intrinsically linked, presenting a major challenge to defining their individual contributions. Moreover, their accurate measurement in both experimental and clinical settings is a technical hurdle. Here, we attempt to deconstruct the contribution of the individual and combined roles of aneuploidy, CIN and ITH to therapy resistance in cancer, and outline emerging approaches to measure and disentangle their roles as a step towards integrating these principles into cancer therapeutic strategy.

## Introduction

Aneuploidy, CIN, and ITH are distinct and important characteristics of tumour cell populations contributing to tumour evolution that are often conflated. In fact, despite their intrinsic mechanistic entanglement, most studies focus on measuring and evaluating just one, or perhaps two, of these three factors to explore their roles in cancer. Aside from the conceptual difficulties in determining individual contributions of aneuploidy, CIN and ITH, additional challenges are presented to the field in the form of inconsistent definitions and technical difficulties in their measurement. In this review, we will outline how aneuploidy, CIN and ITH are implicated in cancer, with a main focus on therapy resistance, although many of the principles and mechanisms are likely to apply to additional aspects of cancer development. We will discuss the difficulties in separating these three factors when interpreting experimental and clinical data and challenges and opportunities to develop standardised and specific measurement methods. Lastly, we will discuss efforts and future opportunities to capitalise on our existing mechanistic knowledge of aneuploidy, CIN and ITH to experimentally disentangle their contributions to therapy resistance.

Box 1: Definitions

**Table:** 

Chromosomal Instability (CIN): An increased rate of chromosomal alteration, which is thought to occur via a myriad of potential dysregulated cellular processes that culminate in aberrant replication, or segregation of chromosomes during mitosis, creating an unequal division of the genetic material between daughter cells. The consequences of CIN range from large alterations in chromosome number and structure (aneuploidy—see below) to smaller, sub-chromosomal alterations termed copy number alterations (CNAs). Aneuploidy: The state of having a number of chromosomes that deviates from a multiple of the normal haploid chromosome number of a given organism. Aneuploidy can be divided into numerical aneuploidy: gains and losses of whole chromosomes which yield a change in the total number of chromosomes and structural aneuploidy: which includes gains, losses, and translocations of parts of chromosomes. Aneuploidy can arise as a result of CIN, though it is also important to note that many of the processes responsible for accurate chromosome segregation are imperfect and normal healthy cells do occasionally mis-segregate chromosomes and generate aneuploid cells, independently of ongoing CIN *per se*. Intratumour heterogeneity (ITH): The high genomic and phenotypic variability found within tumours due to the combined effect of genomic instability and selection pressures, such as chemotherapy.	

## Tumour evolutionary processes connect aneuploidy CIN and ITH

Though this review focusses primarily on the intertwined concepts of aneuploidy, CIN and ITH, each of these are also intrinsically linked to tumour evolutionary processes. We therefore briefly outline the major principles of tumour evolution to place these factors in context, but note that the subject of tumour evolution is extensively reviewed elsewhere (Davis et al. 2017; Black and McGranahan 2021; Vendramin et al. 2021).

Since a landmark 1976 paper hypothesising that tumour development is an evolutionary process to which Darwinian concepts of variation, heredity and selection can be applied (Darwin 1859; Nowell 1976), a range of tumour evolutionary models have been proposed, including linear, branching, neutral and punctuated/macroevolution (Vendramin et al. 2021). Subclonal tumour populations are thought to compete for limited resources, whilst facing fluctuating endogenous and exogenous selective pressures (Greaves and Maley 2012; Vendramin et al. 2021). In contrast, macroevolution is a non-Darwinian theory of tumour evolution positing that large genomic changes form evolutionary ‘jumps’ within a short period of time, facilitated by CIN, aneuploidy and chromothripsis (Vendramin et al. 2021) rather than the progressive action of selective pressures on existing mutations proposed by Darwin. These alterations driven by CIN are arguably more important than point mutations in the development and maintenance of ITH (Andor et al. 2016; Jamal-Hanjani et al. 2017; Turajlic et al. 2018). The aforementioned evolutionary models of cancer are likely not mutually exclusive and may instead occur simultaneously or vary within and between tumours. Of note for this review, aneuploidy, CIN and ITH observed at cancer diagnosis will have been shaped by evolutionary processes, and in turn, tumour evolution dynamics will also be influenced by these three factors.

## Challenges in defining and measuring aneuploidy, CIN and ITH

### Aneuploidy measurement and definition

Aneuploidy has been recognised as a hallmark of tumourigenesis for over a century (Boveri 1902), with some degree of aneuploidy present in 90% of solid tumours and 50% of haematopoietic cancers (Taylor et al. 2018; Replogle et al. 2020), and is more common than any specific gene mutation in cancer. For example, the presence of a trisomy of chromosome arm 1q occurs in 60% of breast cancers, whilst the most common mutated gene, PIK3CA, is only altered in 39% of these tumours (Vasudevan et al. 2021). A well-known, though poorly understood, feature of cancer aneuploidy is the presence of recurrent patterns of chromosome gains and losses, which are frequently characteristic of specific cancer types (Davoli et al. 2013; Taylor et al. 2018; Ben-David and Amon 2019).

The term aneuploidy can refer to whole chromosome changes (numerical aneuploidy) or to the loss or gain of sub-chromosomal regions (structural aneuploidy) (Zack et al. 2013; Taylor et al. 2018). Chromosomal arm alterations (CAAs) have been a popular measure of aneuploidy in many whole genome sequencing (WGS)-based cancer genomics studies. The use of whole chromosome arms as the definition of structural aneuploidy might still be useful and appropriate, yet it is important to recognise; this was defined at a time when the mechanisms for studying karyotypic variation were largely microscopy-based, where identifying a loss of a chromosome arm was the highest resolution possible. However, as the tools for measurement of aneuploidy have evolved from low-resolution microscopy to higher-resolution sequencing-based methods, the definition has expanded to include sub-chromosomal arm alterations (Ben-David and Amon 2019). Indeed, more recent studies (Smith and Sheltzer 2018; Shukla et al. 2020) have chosen to use copy number alterations (CNAs) instead of chromosome arm aneuploidy, making the argument that an arm loss in chromosome 18 is significantly smaller than a chromosome 2 arm loss. Conversely, if a loss the size of an arm of chromosome 18 was to happen within chromosome 2, it would not be considered an aneuploidy by the CAA definition, and thus, its significance would not be included in many aneuploidy studies. Newer methods to detect sub-chromosomal aneuploidy inclusive of smaller CNAs range from genomic array methods such as comparative genomic hybridisation (CGH) and SNP arrays, to bulk tumour sequencing data (e.g. whole genome, or whole exome sequencing) and, more recently, single cell sequencing (see below). Quantitative measures derived using these methods include metrics such as total CNA burden (Hieronymus et al. 2018), fraction of genome altered (FGA) (Zhou et al. 2019) and weighted genome integrity index (wGII) (Endesfelder et al. 2014) among others. Note that these metrics strictly measure the extent of aneuploidy from a static timepoint, yet are frequently used to represent CIN (see below).

### CIN measurement and definition

Whilst CIN is a major driver of aneuploidy and the two are therefore intrinsically linked at a mechanistic level, we must draw a conceptual distinction between them: aneuploidy is a karyotypic *state*, and chromosomal instability (CIN) refers to the *rate* of ongoing chromosome segregation errors over consecutive cell divisions (Lengauer et al. 1998). As outlined in Fig. 2 and reviewed elsewhere (Funk et al. 2016; McClelland 2017; Tijhuis et al. 2019), CIN can arise from multiple potential drivers, including replication stress, or errors in the organisation or segregation of chromosomes. An extreme manifestation of CIN commonly observed in cancer is whole genome doubling (WGD), in which the full genome of a cell is doubled, for example, from a diploid to tetraploid state. Though many drivers of cancer CIN have been proposed, it is likely that the majority of true disease-driving CIN mechanisms are yet to be elucidated, due to major difficulties in detecting and analysing CIN from tumours. Like aneuploidy, CIN is highly prevalent across many cancers, with indications of its presence in 60–80% of human tumours (Carter et al. 2012). Furthermore, cancer-derived cell lines show a much higher rate of CIN than normal, non-transformed cells, at around one chromosome mis-segregation event per 3–10 cell divisions (Thompson and Compton 2008; Bakhoum et al. 2009; Burrell et al. 2013; Laughney et al. 2015; Tamura et al. 2020).

Large chromosomal alterations usually pass through a step of visible mis-segregation during mitosis, providing quantifiable CIN markers (though some chromosome mis-segregation events may be unobservable by microscopy). These markers include lagging anaphase chromosomes, anaphase and ultra-fine bridges, and micronuclei and are often used to experimentally measure CIN rates in vitro (see Fig. 2) (Cimini et al. 2001, 2003; Cimini et al. 2002; Thompson and Compton 2008; Bakhoum et al. 2009, 2011; Kabeche and Compton 2013; Salgueiro et al. 2020). Whilst detecting mitotic errors is technically very challenging in tumour samples, immunohistochemistry (IHC) has been employed to score micronuclei (and therefore infer CIN) in clinical samples with some success (Agustinus et al. 2023). CIN can also be indirectly measured in growing clonal cell populations using fluorescence in situ hybridization (FISH) probes directed against specific centromeres to measure the numerical variation between and within individual clonal populations (Lengauer et al. 1998; Burrell et al. 2013; Tamura et al. 2020). However, this method is very laborious and requires a highly proliferative tumour type to visualise sufficient mitotic cells to score CIN rates (Lynch et al. 2022). Moreover, most smaller sub-chromosomal alterations would not be detectable using microscopy.

Methods to infer CIN from clinical samples have therefore been largely indirect: one such method, the CIN70, infers a signature of CIN from the expression of specific genes consistently associated with aneuploidy across populations of tumour samples. CIN70 was one of the first widely adopted CIN scoring methods, which attempted to address the difficulty of inferring CIN, a rate, from only a single timepoint (and sample) without comparison to another (Carter et al. 2006). However, a subsequent study showed that the CIN70 is not an accurate predictor of CIN and instead highly correlates with cell proliferation rates in both chromosomally stable and unstable cell lines (Sheltzer 2013). This study proposed an alternative proliferation-independent score, HET70, which defines the degree of cell-to-cell karyotypic heterogeneity. However, this still does not allow for direct observation of CIN. Furthermore, neither the CIN70 nor the HET70 correlated with ongoing CIN in cell lines induced to undergo CIN (Lynch et al. 2023). In addition to technical barriers, the CIN field suffers from a lack of a standardised scale of CIN rates: studies frequently refer to spontaneous or induced ‘increases’ in CIN rate, or the comparative impacts of ‘low’ vs ‘intermediate’ vs ‘extreme’ CIN, yet no single or standardised quantitative measure of CIN has emerged (Lynch et al. 2022). Such changes in CIN rates are generally discussed relative to a given researcher’s own data (Tijhuis et al. 2019). Not only does this prevent clarity and reproducibility of data, but it also fundamentally impedes the clinical validation of CIN as a prognostic or predictive biomarker, despite its obvious importance and prevalence. A recent new measure of CIN, mis-segregations per diploid division (MDD) has recently been proposed which may provide a unified measure, though this metric is limited to whole, or large chromosomal alterations and will exclude smaller, sub-chromosomal alterations (Lynch et al. 2023).

### ITH measurement and definition

Early tumourigenesis relies heavily on early driver mutations which are usually present in the whole tumour population (‘clonal’) (Hanahan and Weinberg 2011; Levine et al. 2019). However, tumours subsequently acquire novel genomic changes in a subclonal manner, with cell populations ‘branching’ off from the original lineage. This produces genomically diverse subpopulations of cells within the same tumour and gives rise to ITH by virtue of additional mutations, or chromosomal alterations occurring after the branching point (Gerlinger et al. 2012). Whilst small-scale genetic alterations such as point mutations clearly contribute to ITH, large scale genomic alterations, such as aneuploidies resulting from CIN, may have a larger impact on cellular phenotypes and drive tumour diversification at a higher rate (Marusyk et al. 2020). For simplicity in this review, we focus primarily on ITH as it relates to CIN, but it is important to note that ITH is the combined result of both CIN and evolutionary selection process (as discussed briefly above).

Whole genome doubling, as well as being a major class of CIN event, can also act secondarily to increase ITH and facilitate tumour evolution, and has thus been termed a macroevolutionary event (Prasad et al. 2022). WGD has been proposed as a means to mitigate the accumulation of detrimental alterations due to instability, such as loss of heterozygosity (LOH) caused by chromosome or sub-chromosomal losses (López et al. 2020; Vendramin et al. 2021; Keuper et al. 2021). LOH involves the loss of allelic variation in a given gene, forcing the cell to depend on a single allele. By producing an additional copy of the remaining allele, WGD creates more permissive conditions for further copy loss (e.g. of essential genes) that may occur following further CIN, aneuploidy and other genomic alteration (Watkins et al. 2020). WGD not only enables the tolerance of these processes, but also appears to enhance them (Dutrillaux et al. 1991; Zack et al. 2013; Dewhurst et al. 2014; Kuznetsova et al. 2015; Boisselier et al. 2018; Prasad et al. 2022). In this sense, CIN is acting not only to increase ITH through the continual generation of heterogeneous aneuploid states, but also creates extreme ploidy changes that permit further diversification via increased tolerance. ITH, if extensive enough, could provide a vast reservoir of genetic or genomic alterations that might promote therapy resistance (see below).

Methods of quantifying ITH have been comprehensively reviewed by Kashyap et al. (2022), though it is important to note that measurement of *genetic* ITH relies heavily on bulk sequencing, including both whole-exome and whole-genome sequencing. The latter has been employed to great success in a recent study which utilized a consensus-based strategy of calling copy numbers and mutations in order to assess ITH. The authors demonstrated that a high percentage of samples contained evidence of distinct subclonal expansion, branching evolution, and positive selection—and, interestingly, variation in mutational processes between distinct subclones (Dentro et al. 2021).

However, measurement of ITH depends not only on the precise meaning of ITH being used by the researcher (e.g. ITH in point mutations, in CNAs, in genome ploidy) but also on the regional scale of the tumour ITH being assessed. For example, ITH may refer to large topological regions of tumours and can be measured by analysing and comparing multiple regions (multi-region sequencing, e.g. Gerlinger et al. 2012). Patient sampling methods are a limiting factor in this process, as comprehensively reviewed by Vishwakarma and McManus (2020). Moreover, the accuracy of tools for patient stratification is impacted by the restricted region of a tumour that is sampled, which cannot provide the spatio-temporal resolution needed to measure genomic heterogeneity or CIN rate throughout the tumour. Profiling of CIN, aneuploidy and heterogeneity from samples removed at initial surgical intervention may also be very different to that of a later disease stage (Hiley and Swanton 2014). Multi-region sampling can certainly improve on these issues, but even this may still underestimate various measures of heterogeneity between cells, due to the difficulty of detecting low frequency variants (Salk et al. 2018). When considering ITH as generated by CIN, or if one is searching for an extremely rare cell or small subclone harbouring a pre-existing resistance phenotype, cell-to-cell ITH becomes extremely important.

## Using single cell sequencing to quantify aneuploidy, CIN and ITH

Recent sequencing advances have provided the next generation of measurement tools that are beginning to allow researchers to more precisely and specifically measure aneuploidy, CIN and ITH. Specifically, the advent of single cell sequencing (SCS) not only allows the fine-grained analysis of aneuploidy, measuring cell by cell chromosome variation across hundreds to thousands of cells (Navin et al. 2011; Wang et al. 2014; Salehi et al. 2021; Funnell et al. 2022), but also improves estimate of CIN rates. Whilst SCS in isolation at a fixed timepoint still cannot directly measure CIN, this is becoming increasingly possible when SCS is combined with phylogenetic/tumour evolution models (Lynch et al. 2022; Kaufmann et al. 2022), or when applying SCS after clonal outgrowth (Bolhaqueiro et al. 2019; our unpublished data). The high throughput capability of this technology is incredibly valuable when considering the time-consuming nature of microscopy-based CIN measurements. A recent paper by Lynch et al. utilized a novel framework of measuring underlying tumour evolutionary processes via SCS to account for the effect of selection when quantitatively measuring CIN (Lynch et al. 2022), validating the approach in cells after paclitaxel induction of CIN and in cancer biopsy and organoid samples. This method also provided a valuable insight that 200 cells is the minimum number required from a human tumour sample, for (≥ 90%) accurate, reproducible CIN quantification. An alternative strategy has also been used to identify evidence of chromosome mis-segregation events from large-scale single cell sequencing of cancer cell lines, whereby clones of cells are identified based on similar CNA profiles, and CNAs differing from the consensus are assumed to be the consequence of recent CIN (Laks et al. 2019; Funnell et al. 2022). Though these approaches have limitations, particularly their inability to account for fluctuations in CIN rates over space or time, they represent a substantial step in the development of standardised CIN quantification as a tool for research and as a prognostic biomarker in patients, which could be fundamental in informing the most appropriate therapeutic strategy. A key remaining challenge is to decipher the mechanistic origin of newly arising aneuploidies and CNAs in order to infer cancer CIN mechanisms.

## The challenge of cancer therapy resistance and the potential roles of aneuploidy, CIN and ITH

Aneuploidy, CIN and ITH have all independently been associated with poor cancer patient prognosis (Kheir et al. 1988; Choma et al. 2001; Walther et al. 2008; Andor et al. 2016; Taylor et al. 2018; Hieronymus et al. 2018; Bakhoum and Cantley 2018; Smith and Sheltzer 2018; Ben-David and Amon 2019; Shukla et al. 2020; Ramón y Cajal et al. 2020; Hua et al. 2020) and it is likely that a unifying reason for this is their individual and/or combined roles in promoting therapy resistance.

Recent decades have seen vast progress in the treatment of primary and, to some extent, metastatic cancer via a range of therapeutic regimens. Chemotherapy is the most common treatment strategy, and many patients see remarkable initial success following this, with partial or complete remission (Urruticoechea et al. 2010; Baskar et al. 2012; Damin and Lazzaron 2014; Khalil et al. 2016; Wang et al. 2019). However, the recurrence of aggressive, metastatic disease is a huge global burden across cancer types (Castells et al. 2012; Delgado-López and Corrales-García 2016; Fink-Neuboeck et al. 2020; Baek and Lee 2020). The development of drug resistance is also a major issue, reported for almost every chemotherapeutic in use (Ramos-Martínez et al. 2021), and poses a formidable threat to cancer patient survival; 80–90% of metastatic cancer patient mortality is attributed directly or indirectly to resistance (Longley and Johnston 2005; Mansoori et al. 2017). Resistance can be specific to one drug or drug class, or simultaneous for various drugs with different mechanisms of action, termed multidrug resistance (MDR) (Ramos-Martínez et al. 2021; Emran et al. 2022).

Generally, therapy resistance is separated into two main classes: intrinsic and acquired. Cancer therapy resistance can be mediated by genetic, non-genetic or tumour microenvironmental changes (Emran et al. 2022), but here, we focus on genetic or genomic-related mechanisms as they relate to aneuploidy, CIN and ITH. Intrinsic resistance is present before treatment begins and reduces the initial efficacy of therapy. It can be attributed to inherent resistance-conferring mutations, or the activation of intrinsic pathways which enable resistance. Conversely, acquired resistance is described as the development of resistance following repeated drug administrations, leading to a gradual reduction in drug potency (Foo and Michor 2014; Wang et al. 2019). This process is exemplified by the ever-diminishing returns provided by the treatment of high-grade serous ovarian carcinoma patients with successive chemotherapy cycles (Christie and Bowtell 2017). Acquired resistance can be driven by the presence of low-frequency resistant subclones that exist prior to treatment and which expand to reform resistant disease during and/or following therapy administration. Alternatively, acquired resistance can be caused by novel genetic or genomic alterations arising and expanding during or after the treatment regime (Thress et al. 2015; Wang et al. 2019; Emran et al. 2022). It is also worth considering that there is evidence that drug resistance can arise from either, or both, intrinsic and acquired resistance within the same disease (Mavrommati et al. 2021). When considering the role of aneuploidy, CIN and ITH in therapy resistance, a fundamental issue is that it has not been possible in any studies to entirely separate the three phenomena, making it difficult to identify specific mechanisms causing resistance. We outline below the potential contributions of each factor to therapy resistance.

## Aneuploidy in prognosis and therapy resistance

In both normal and cancer cells, aneuploidy has numerous consequences, impacting metabolism, protein homeostasis, stress response genes and cell proliferation (comprehensively reviewed in Chunduri & Storchová (2019). As a consequence, aneuploidy can induce cell cycle arrest in normal cells and reduce their fitness by an estimated 6–30% (Bakhoum and Landau 2017). It is worth noting that more recent studies suggest that some of the detrimental effects on cell cycle progression observed in earlier work may stem from inadvertent activation of the DNA damage response as a consequence of methods used to induce aneuploidy. For example, p53-dependent cell cycle arrest was only observed in cells undergoing structural aneuploidy events, and not as a result of whole chromosome aneuploidy (Soto et al. 2017). However, most aneuploidies arising during meiosis and embryogenesis are lethal, and those that are viable (such as the trisomy of chromosome 21, which causes Down Syndrome) usually induce substantial disabilities (Siegel and Amon 2012). In contrast, aneuploidy appears to confer a fitness advantage in many cancers. This contradictory relationship between the beneficial and detrimental effects of aneuploidy on cell fitness is described as the aneuploidy paradox, and the net impact of aneuploidy is thought to depend on the environmental context, cell type, affected chromosomes and mechanism of origin (Weaver et al. 2007; Sheltzer et al. 2017; Vasudevan et al. 2020).

There are several proposed rationales for why aneuploidy confers an advantage to cancer cells which can promote cancer cell fitness and facilitate the development of therapy resistance. These are not necessarily mutually exclusive and could act in combination(s).

### The tumour promoting impact of gene dosage alterations

Benefits provided by aneuploidy could result from the amplification or deletion of key genes, contained within genomic regions affected by aneuploidy (and therefore present in increased, or decreased gene copy number), which increase cells’ ability to cope with environmental stressors, including therapy. Substantial evidence supports the view that increased oncogene and reduced tumour suppressor gene dosage drive the fitness benefit conferred by aneuploidy, especially in genes demonstrating an altered phenotype when either one or three copies are present—haploinsufficiency and triplosensitivity, respectively (Beroukhim et al. 2010; Jones et al. 2010; Gordon et al. 2012; Davoli et al. 2013; Smith and Sheltzer 2018; Schukken and Sheltzer 2022). Another set of studies revealed that, when treating patient-derived cells and xenografts with targeted drugs inhibiting specific oncogenes, parallel and convergent amplifications occurred in specific gene regions that enabled treatment resistance. For example, in studies of EGFR-targeted inhibition, resistance emerged due to the rapid selection of cells with aneuploidies or copy number amplifications in either the EGFR gene or of other relevant downstream genes, allowing chemoresistance for this targeted treatment strategy (Engelman et al. 2007; Bean et al. 2007; Pavelka et al. 2010; Chen et al. 2015). Similarly, lung and melanoma patient-derived xenografts treated with inhibitors of the MAPK signalling pathway developed amplifications of the BRAF gene, which allowed the bypass of the targeted oncogene inhibition (Xue et al. 2017). These amplifications arose independently in separate subclones, thus maintaining a level of intratumoural genetic heterogeneity.

Recent studies testing the contribution of CIN and aneuploidy to therapy resistance showed that cancer cell lines exposed to a variety of chemotherapeutic agents had increased proliferation capacity when pre-treated with transient CIN-inducing agents (Rutledge et al. 2016; Ippolito et al. 2021; Lukow et al. 2021). Chemo-resistant populations displayed recurrent aneuploidies specific to the cell line and chemotherapy combination, suggesting that specific chromosomal gains and losses provide increased fitness, possibly due to amplification of specific genes involved in resistance. Ippolito and colleagues investigated the exact molecular mechanism by which new, recurrent aneuploidies can drive resistance in non-small cell lung cancer cells. They found that a cell line which developed resistance to the topoisomerase I inhibitor topotecan displayed both proteomic and transcriptomic upregulation of an efflux pump (BCRP); however, the gene encoding this pump was not itself encoded on a recurrently aneuploid chromosome. Instead, the authors discovered that a subunit of the stress kinase p38 (*MAPK13*) that was present within a recurrently aneuploid (gained) chromosomal region in resistant cell populations was linked to upregulated expression of BCRP (Ippolito et al. 2021). Their work beautifully exemplifies how specific aneuploid states can confer resistance, and how in-depth functional studies are required to dissect the potentially complex route from aneuploidy patterns to a functional role in drug resistance. In this instance, a combination of transient CIN, the resultant genomic heterogeneity and the selection of specific aneuploidies facilitated the development of the preferential aneuploid states, demonstrating the difficulty in assigning resistance drive to any individual contribution alone.

Not all aneuploidies, however, will result in a phenotypic change. Even though copy number gains generally result in increased gene expression, around 20% of the encoded proteins are subsequently subject to dosage compensation, which is especially common in genes encoding subunits of protein complexes (Dephoure et al. 2014; Gonçalves et al. 2017; Schukken and Sheltzer 2022; Cheng et al. 2022). This seems to be a highly conserved mechanism in eukaryotes, which relies on post-translational and post-transcriptional degradation to tolerate the detrimental effects of aneuploidy (Gonçalves et al. 2017; Senger and Schaefer 2021; Schukken and Sheltzer 2022). Interestingly, there is evidence that copy number alterations are prognostic regardless of their effect on gene expression (Smith and Sheltzer 2018).

### Systemic ‘tolerant’ state

The continued proliferation of aneuploid cells is likely to require genetic mutation or altered expression of certain proteins which can increase the tolerance to aneuploidy by altering pathways which would normally cause cell cycle arrest and senescence, such as *TP53* (Thompson and Compton 2008; Torres et al. 2010; López-García et al. 2017; Soto et al. 2019). A secondary consequence of this ‘aneuploidy tolerant state’ may be the concomitant tolerance to cancer therapies. Aneuploid cells may be better primed for environmental challenges due to their already increased expression of stress response pathways (Dephoure et al. 2014; Rutledge et al. 2016; Zerbib et al. 2023). The impact of aneuploidy in the transcriptional landscape has been extensively analysed, with several studies identifying chromosome-independent aneuploidy transcriptional signatures (Sheltzer et al. 2012; Sheltzer 2013; Zerbib et al. 2023). In this latter study, this signature was mostly characterised by the upregulation of DNA damage repair pathways, RNA metabolism and proteotoxic stress tolerance pathways; and the downregulation of transcriptional signatures associated with cell cycle and drug metabolism (Zerbib et al. 2023). This same study explored the general dependence of aneuploid cells on the RAF/MEK/ERK signalling pathway (associated with oncogenesis in many cancers) and linked c-Raf enzyme activity to the decreased sensitivity of aneuploid cells to DNA damage-inducing chemotherapy, thus providing a chromosome identity-independent mechanism for chemo-resistance in aneuploid cells. Another study using colorectal cancer cell line HCT116 engineered to harbour specific trisomies showed decreased chemosensitivity to paclitaxel, a microtubule stabilising drug used in cancer treatment, regardless of which chromosome was present in an extra copy (Replogle et al. 2020). This suggests that resistance was driven by the aneuploid state rather than the increased copy number and expression of specific genes. Since aneuploidy can reduce proliferation rates, the authors hypothesised that this may confer protection from paclitaxel treatment. Indeed, further experiments equalising proliferation rates between the diploid and trisomic cells reduced the differential sensitivity to the treatment, suggesting that the reduced proliferation rather than aneuploidy *per se* conferred chemoresistance (Replogle et al. 2020).

### Aneuploidy-induced increase in genomic and chromosomal instability

Whilst it is well-established that CIN drives aneuploidy, there is increasing evidence that aneuploidy can in turn enhance the rate of CIN (Passerini et al. 2016; Garribba et al. 2023), which may represent an important contributor to the adaptive advantage conferred by aneuploidy in cancer. Early yeast studies showed that aneuploidy can induce high rates of chromosome mis-segregation (Sheltzer et al. 2011), potentially via the imbalance of important mitotic checkpoint genes (Zhu et al. 2012). The addition of extra chromosomes in human cells also induces chromosomal instability, with one landmark study demonstrating increased replication stress and chromosome segregation errors in trisomic and tetrasomic cells compared to their chromosomally stable parental cells, ultimately traced back to dysregulated replicative machinery levels (Passerini et al. 2016). Similarly, there is some evidence of increased rates of CIN arising in cells from people with congenital whole chromosome aneuploidies (Valind et al. 2013), which may underlie the increased risk of developing particular malignancies observed in various congenital aneuploid conditions, as outlined in detail in a review by Vargas-Rondón et al. (2017). A more recent study demonstrated that the gain of extra chromosomes (but not chromosome loss) in human p53-deficient cells appeared to increase CIN levels (Hintzen et al. 2022), and it has been proposed that the increased burden on protein turnover machinery, induced by chromosome gains, may underlie this effect.

Overall, aneuploidy has multiple potential routes to promote therapy resistance, again highlighting the difficulty of narrowing down the precise mechanism responsible.

## CIN in prognosis and therapy resistance

CIN is considered to act as a biological bet-hedging system during tumour evolution (Kussell and Leibler 2005; Lukow and Sheltzer 2022), and many studies have highlighted the relationship between elevated CIN rate and the development of therapeutic resistance in cancer (Kaelin 2005; Penner-Goeke et al. 2017; Kim et al. 2018). Studies have shown that high CIN rates are associated with intrinsic drug resistance across many cancers (Lee et al. 2011; Burrell and Swanton 2014), and that the presence of CIN can inform both chemoresistance and drug sensitivity in response to particular therapeutics (Bartlett et al. 2010; Munro et al. 2012). One such example is that measures of baseline CIN appear to predict response to the chemotherapeutic drug paclitaxel (Swanton et al. 2009; Janssen et al. 2009). The major caveat for many of these studies is that CIN metrics used often measure aneuploidy, rather than rates of CIN, suggesting that a careful re-examination of the relationship between CIN and therapy resistance using newer, improved metrics of CIN may shed more light on this important association.

### CIN as a driver of aneuploidy and ITH

As discussed above, work from the Sheltzer and Santaguida laboratories found that CIN-induced resistant cell lines reproducibly acquired specific aneuploidies (Ippolito et al. 2021; Lukow et al. 2021). The authors suggested that this could be evidence of CIN driving a converging positive selection of recurrent aneuploidies. In this instance, CIN’s role was temporary and simply provided the initial substrate of a heterogeneous aneuploid cell population. However, another study tested how accurately various computational models predict evolution of subclonal copy number alterations that arise after tumour diversification, using the most recent common ancestor of each tumour as a starting point (Watkins et al. 2020). The most accurate model found was one in which positive selection of oncogenes and negative selection of tumour-suppressor genes was favoured, with this study also finding evidence of parallel evolutionary events disrupting the same genes in separate subclones (Watkins et al. 2020). The authors suggested that these results may indicate a role for CIN across the stages of tumour evolution and that the CNA landscape may be shaped by ongoing selection enabled by CIN.

In line with this hypothesis, a recent study detected evidence of ongoing CIN driving resistance to MAP kinase inhibitors in metastatic melanoma patient samples. Elevated non-homologous end joining (NHEJ) appeared to drive the amplification of resistance driver genes via complex genomic rearrangements and extra-chromosomal DNA (Dharanipragada et al. 2023). Excitingly, limiting CIN (inhibiting NHEJ using DNA-PK_CS_ inhibitors) during therapy treatment of melanoma cell lines and patient-derived xenograft (PDX) models prevented the amplification of resistance driver genes and delayed the development of resistance to MAP kinase inhibitors (Dharanipragada et al. 2023). As newer technologies allow an increased molecular understanding of ongoing CIN mechanisms in tumours, and particularly during therapy, it is tempting to speculate that similar approaches to limit specific CIN mechanisms could delay the onset of resistant diseases in many other cancer types. In our opinion, this study also demonstrates the importance of considering the full range of genomic alterations that can be caused by CIN, in addition to large scale alterations such as whole chromosome, or chromosome arm aneuploidy, since even small-scale genomic alterations resulting from CIN are capable of providing strong resistance phenotypes.

Many cancers have an ‘addiction’ to specific oncogenes, which can be highly useful in enabling more direct treatment using targeted therapies such as kinase inhibitors. Often this treatment is initially very effective, but there is an inevitable resistance development and subsequent tumour recurrence. CIN may well play a role in this by removing the dependence of cancer cells on specific oncogenic events via karyotype diversification (Bakhoum and Landau 2017; Bronder and Bakhoum 2020). A landmark study used a mouse model of lung cancer driven by the KRAS oncogene and induced periods of CIN via Mad2 overexpression to examine this. Without treatment, tumours with induced CIN developed more slowly than those without. However, after KRAS inhibition, CIN-induced tumours were significantly more likely to relapse after treatment than those without CIN induction (Sotillo et al. 2010). Similarly, in a mouse KRAS^G12D^ breast tumour model, elevated Mad2 expression enabled increased CIN before and during tumour growth and promoted the development of oncogene-independent subclones (Rowald et al. 2016). Furthermore, a recent study showed that CIN-induced breast tumours which had relapsed and were now KRAS-independent had recurrent novel subclonal copy number amplifications of another oncogene, *MET*, which encodes the receptor tyrosine kinase cMET (Salgueiro et al. 2020). This amplification resulted in increased cMET mRNA and phosphorylated cMET protein levels and the now KRAS-independent tumours were shown to be dependent on cMET for their growth and viability. A small number of control tumours (in which CIN was not experimentally induced) were also able to persist following KRAS inhibition. Interestingly, these tumours also showed spontaneous elevated CIN rates similar to those of the induced tumours, as well as the aforementioned MET/cMET increase. These results suggest that elevated CIN is a pre-requisite for therapy resistance in this experimental setting, and that tumours can modulate CIN levels to achieve resistance, though the driver(s) of increased CIN remain elusive in this instance.

### CIN’s role as a process

In the examples above, CIN’s role is to provide a diverse substrate of aneuploid states, but there is also evidence that the CIN process itself provides an alternative potential route to promote therapy resistance. One example is chromothripsis, the shattering and reassembly of one or a small number of chromosomes that allows multiple rearrangements to occur in close proximity (Cortés-Ciriano et al. 2020), and which may be a key mechanism to promote therapy resistance (Lee et al. 2017; Shoshani et al. 2020). Chromothripsis frequently occurs in chromosomes that are mis-segregated or sequestered in micronuclei (Huang et al. 2011; Stephens et al. 2011; Crasta et al. 2012; Notta et al. 2016). These micronuclei may also rupture and release their genomic DNA into the cytosol, as demonstrated in several studies (Hatch et al. 2013, Mackenzie et al. 2017, Liu et al. 2018). The presence of cytosolic DNA activates the cGAS-STING (cyclic GMP-AMP synthase-stimulator of interferon genes) pathway which induces the transcriptional upregulation of epithelial-to-mesenchymal transition (EMT)-related genes, promoting migratory and invasive cellular properties, via non-canonical activation of NF-κB signalling (Sun et al. 2013; Abe and Barber 2014; Bakhoum et al. 2018). Furthermore, direct suppression of ongoing CIN markedly reduced metastasis, even when high aneuploidy is stably maintained, providing evidence that the *process*, as well as the *outcome* of CIN may be important for cancer progression (Bakhoum et al. 2018). Though this study focused on metastasis, it is possible that additional downstream impacts of CIN, micronucleus formation and cGAS-STING signalling might also be important in promoting therapy resistance. Critically, this experimental setup provides the opportunity to examine this hypothesis in the future, due to the ability to disentangle ongoing CIN from pre-existing aneuploidy and ITH by the use of CIN—reducing manipulations in the tumour initiating cells (also see below).

## ITH and tumour evolution in prognosis and therapy resistance

**Fig. 1:**
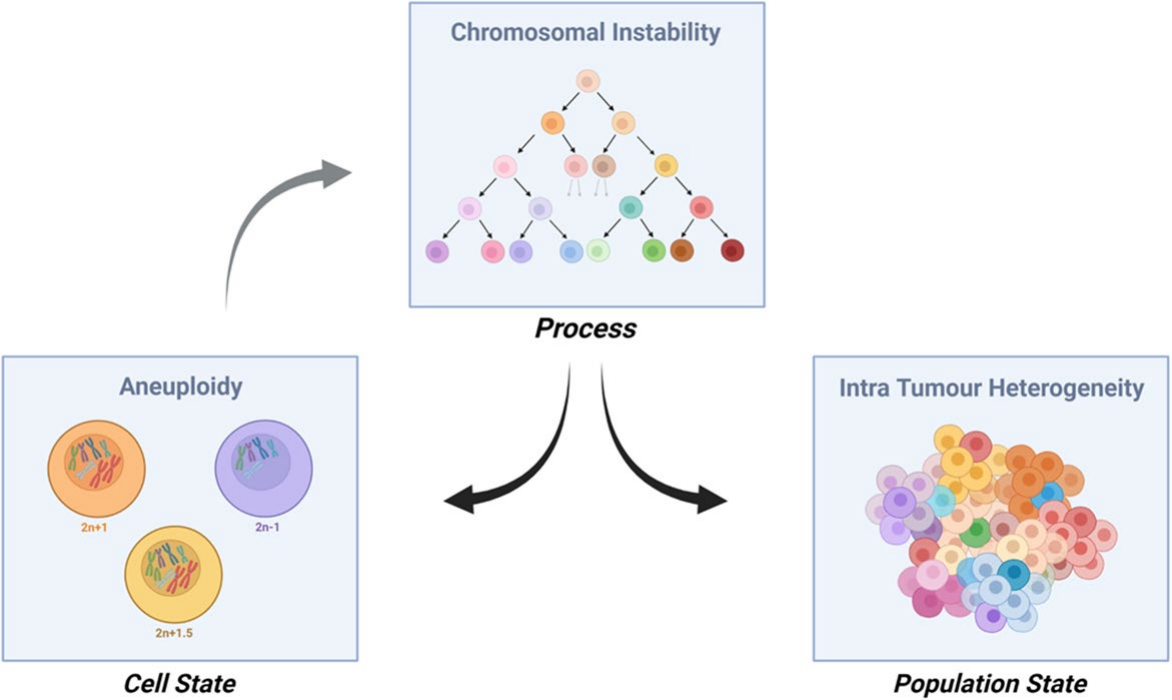
Chromosomal instability (CIN) is a process that generates heterogeneous aneuploid cell populations. It is important to note that aneuploidy and intra tumour heterogeneity (ITH) describe a cellular and a population state, respectively, whilst CIN is the process that can generate them. As indicated by the grey arrow, there is evidence that aneuploidy, even when generated via non-CIN mechanisms, can itself trigger CIN. Thus, these three factors are tightly interconnected and interdependent

**Fig. 2:**
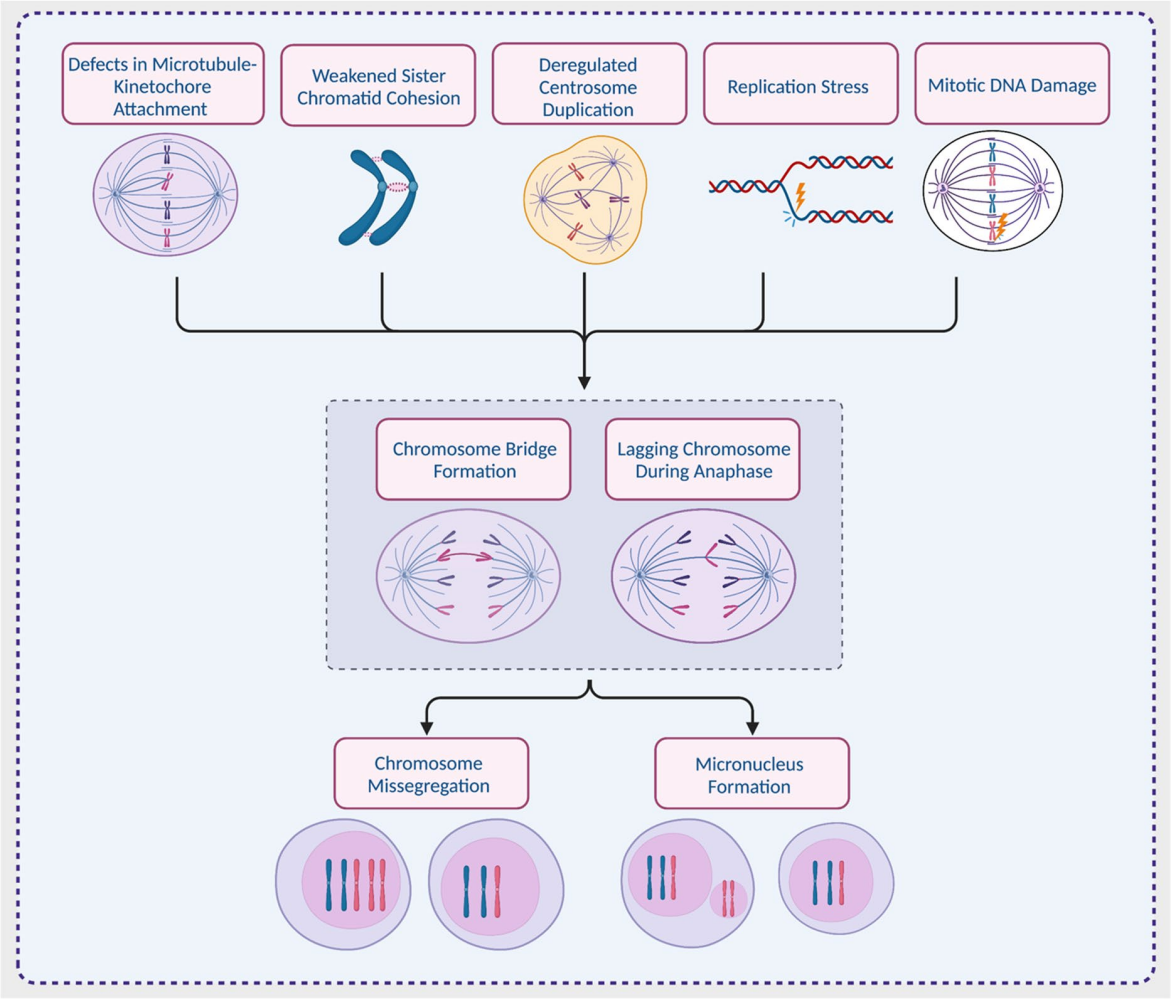
Cellular causes and consequences of CIN. Multiple defective cellular processes, including errors during chromosome replication and organisation, can result in lagging chromosomes or chromatin bridges during anaphase and can also result in the encapsulation of lagging chromosomes in small extranuclear bodies, known as micronuclei. The mis-segregation of chromosomes produces daughter cells with numerical and/or structural aneuploidy. Created with BioRender.com

**Fig. 3:**
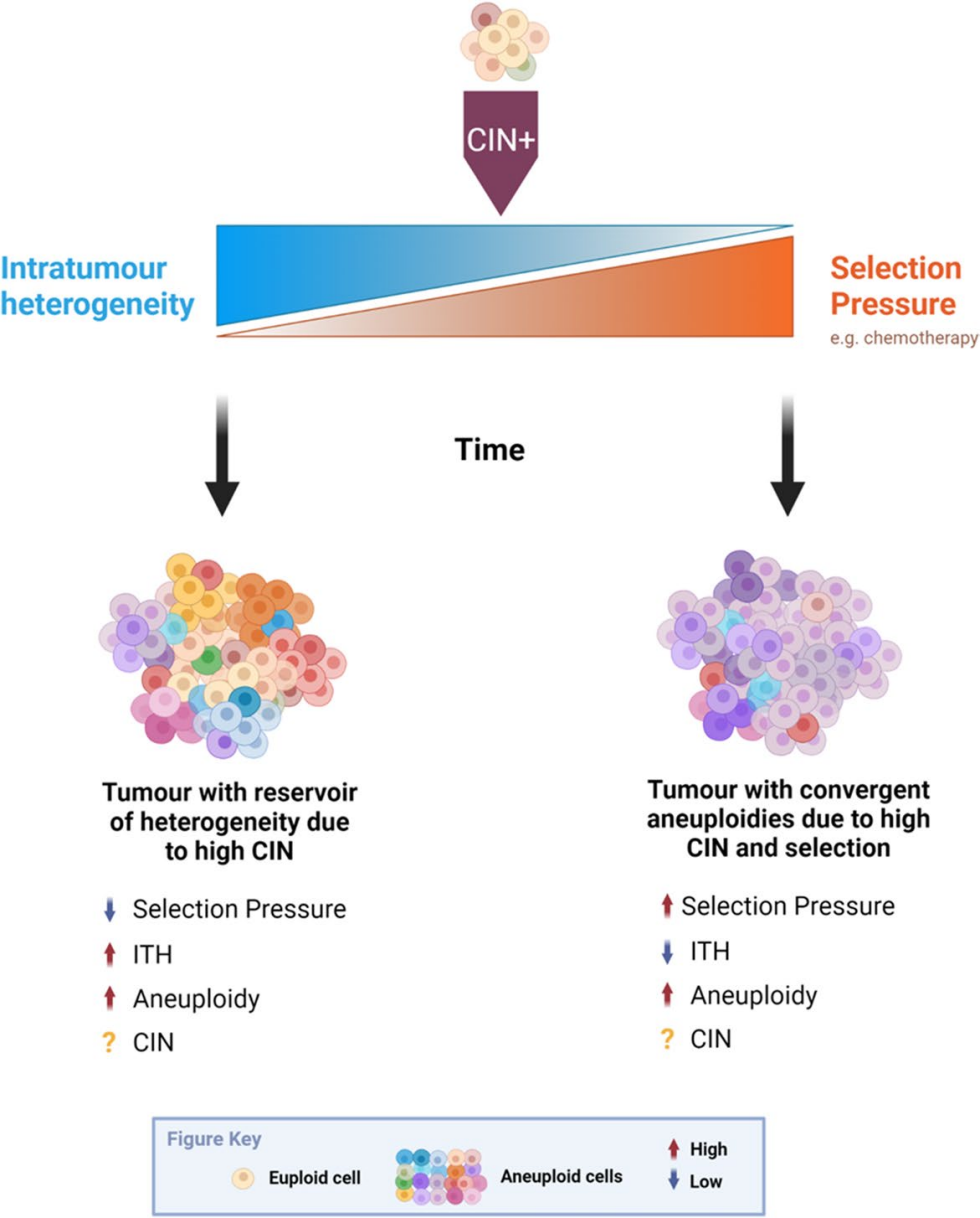
The interplay between CIN and ITH. Intratumour heterogeneity (ITH) is a variable strongly influenced by both CIN and evolutionary selection processes. When CIN rates are unknown, low ITH could represent either low CIN, or high selection, or a combination thereof

The net result of CIN is not just aneuploidy, but heterogeneous aneuploidy, giving rise to ITH (see Fig. 1 and Fig. 2). ITH provides a substrate for evolutionary processes, which in turn shape the karyotypic landscape (Fig. 3). Because a more heterogenous population is more likely to enable evolution toward a therapy resistant state, ITH *per se*, even independently of ongoing CIN, is likely to be an important driver of therapy resistance in cancer, as discussed above, and can be used to predict cancer patient prognosis (Andor et al. 2016). In chronic lymphocytic leukaemia (CLL), the presence of subclones with driver mutations was shown to be an independent risk factor for disease progression (Landau et al. 2013). Similarly, Espiritu et al. (2018) demonstrate this utility in their study of localized prostate tumours, in which they found that polyclonal tumours were significantly more likely to undergo relapse after treatment than their monoclonal counterparts, and that increased heterogeneity in tumour subpopulations correlated with an increased likelihood of metastasis. However, it is also noted that the most aggressive polyclonal tumours were also characterised by higher levels of genomic instability, highlighting the interplay between these factors (Espiritu et al. 2018). The role of ITH in predicating therapy resistance is also illustrated by studies demonstrating multiple distinct mechanisms of resistance to the same selective pressure. For example, during the development of resistance to BRAF inhibition in melanoma, the MAP kinase pathway was re-activated via several different routes such as RAS mutations, mutant BRAF amplification and alternative splicing. PI3K-PTEN-AKT-upregulation was also described, and in 20% of patients, the authors interestingly detected at least two different resistance mechanisms (Shi et al. 2014). In other studies examining resistance mechanisms in response to RAF inhibition, P13K inhibition or EGFR antibody therapy, mutations tended to affect the same therapy-specific genes (e.g. KRAS in response to EGFR blockade), but via distinct mutations or alterations (Bettegowda et al. 2014; Misale et al. 2012; Juric et al. 2015). The numerous distinct routes to therapy resistance observed in these and similar studies highlights the extraordinary ability of heterogeneous tumour cell populations to evade therapy, though it is still not clear in most cases whether these alterations were present prior to, or developed during, therapy administration. These studies also focussed mainly on small-scale genomic alterations such as point mutations or specific gene amplifications, whereas tracking the impact of heterogeneous chromosomal alterations presents perhaps a greater challenge since the functional significance of the majority of cancer aneuploidies remains unclear.

Although ITH in terms of chromosomal alterations can be directly measured in tumours with ever greater precision (for example at cell-cell resolution), lack of knowledge regarding the concomitant processes of CIN and/or evolutionary selection present a challenge to a clear understanding of the role of ITH in therapy resistance. Indeed, for the majority of cancers, and as illustrated in the examples above, it remains unresolved whether the heterogeneity in genomic alterations present at the point of treatment initiation, or the process of CIN during and in response to treatment, are more important in the development of chemo-resistance. Whilst ITH represents a state of high diversity within a tumour and provides a rich substrate for selective pressures to act upon, CIN is likely concomitant, making it difficult to separate the two factors when inferring ITH as a predictor of prognosis. ITH is therefore so exquisitely entangled with aneuploidy, CIN and tumour evolution, it perhaps presents the most difficult of the three factors discussed in this review for which to clarify mechanistic contributions to therapy resistance. Developing a more thorough understanding of the contributary processes to ITH and how to monitor and predict this in patients would have a large impact on cancer therapy.

## How can future research disentangle CIN, aneuploidy and ITH?

Increased mechanistic knowledge of the drivers of aneuploidy, CIN and ITH may allow researchers to manipulate each factor independently and establish a deeper understanding of their individual roles in the disease state. Bakhoum and colleagues were able to do this experimentally (Bakhoum et al. 2018) in transplantable metastatic tumour models of human or mouse triple-negative breast cancer and lung adenocarcinoma. Having shown that elevated CIN promoted metastasis, the authors then used direct suppression of CIN (via KIF2C overexpression—impacting MT stability) to assess whether this same effect was observed when previously chromosomally unstable cells now stably maintained their aneuploid karyotypes. Highly aneuploid cells with CIN suppression showed a marked reduction or delay in metastasis. This experimental design thus enabled the authors to determine that it was an element of the ongoing CIN process itself, rather than the karyotypic consequences of this in isolation, driving metastasis in this model.

Elevated microtubule assembly rates were shown to promote CIN in colorectal (Ertych et al. 2014) and high-grade serous ovarian (Tamura et al. 2020) cancers. Knowledge of the cellular mechanisms promoting this phenomenon allowed the Bastians laboratory to test the impact of reducing CIN in tumour formation rates (Ertych et al. 2014). Unexpectedly, lowering CIN promoted tumour growth in mouse sub-cutaneous injection models, potentially due to a lower negative fitness. The ability of lower CIN tumours to develop therapy resistance was not tested using this experimental set up but would be very interesting to address in the future.

Another approach to experimentally suppress CIN levels is to reduce replication stress, a cellular process also thought to be an important driver of CIN, as shown in colorectal cancer (Burrell et al. 2013), non-small cell lung carcinoma (Venkatesan et al. 2021) and high-grade serous ovarian cancer (Tamura et al. 2020). Replication stress involves the slowing or stalling of replication forks and can induce varied cellular phenotypes including increased prometaphase DNA damage and elevated chromosome segregation errors (Gagou et al. 2010; Zeman and Cimprich 2014). Exogenous nucleosides are used to ‘rescue’ this replication stress and can reduce CIN levels in CRC (Burrell et al. 2013) and HGSOC (Tamura et al. 2020). Thus, a variety of experimental tools are available to investigate the effect of reducing CIN on the development and maintenance of chemoresistance and determine the importance of ongoing CIN in this crucial element of tumour evolution. However, there are also caveats with these approaches: reducing CIN will also reduce aneuploidy and ITH, depending on both the timescale of the experiment and the model used. Additionally, there may be additional consequences of reducing replication stress or aberrant microtubule dynamics that would alter the outcome of such experiments independently of altered CIN rates.

Aneuploidy has frequently been induced by transient interference with mitotic checkpoints (e.g. via reversine or Mps1 inhibition) which generate aneuploid populations with heterogeneous, complex karyotypes and potentially multiple gains and losses in each cell (Sotillo et al. 2010; Foijer et al. 2014; Santaguida et al. 2017). Using such methods allows for the study of the acute effect of aneuploidy, but it may also promote aneuploidy-induced chromosomal instability, making it difficult to untangle whether the observed phenotypes are due to the specific aneuploidies gained, the aneuploid status itself, aneuploidy-induced CIN or ITH. Fortunately, new experimental models for the isolated study of aneuploidy as a contributor to cancer phenotypes are becoming increasingly available. Methods to induce whole and partial chromosomal aneuploidies include the induction of single targeted trisomies or monosomies via techniques such as microcell-mediated chromosome transfer (Rutledge et al. 2016; Sheltzer et al. 2017; Vasudevan et al. 2020) or targeted chromosome mis-segregation (Truong et al. 2023; Tovini et al. 2023; Bosco et al. 2023). New methods for the generation of chromosome arm deletions, which are increasingly recognised as more potent predictors of disease severity, have also been emerging—one such example was developed via engineering breaks with the insertion of artificial telomeres, using a CRISPR-Cas9 system (Taylor et al. 2018). The reverse approach has also been pioneered by the Sheltzer laboratory, who restored the normal copy number of chromosomal arms present in trisomy in cancer cell lines (Girish et al. 2023), thus allowing the study of the importance of specific recurrent cancer aneuploidies in isolation from CIN or ITH.

## Conclusion

The interplay between chromosomal instability, aneuploidy and ITH in the context of cancer evolution, and particularly therapy resistance development is a complex one. Distinguishing the contribution of each of these elements is a long-standing challenge, and many studies have focused on one or two elements, relying on asserting findings about the others via inference. However, to understand their interplay at a level compatible with combating cancer therapy resistance, we reason a holistic approach is required that includes accurate measurement and definition of each factor, alongside approaches to assign the importance of each during the progression of cancer and during treatment. Additionally, a major hurdle for determining the impact of aneuploidy, CIN and ITH on cancer progression and therapy resistance has been the lack of accurate measurement of each parameter from clinical tumour samples. For example, the majority of studies classify aneuploidy by measures such as fraction of genome altered, or number of chromosomes or chromosome arms in aneuploid states. However, this is usually from bulk (population) analyses and will detect only chromosomal alterations present in relatively large clones or subclones. Current technologies allow for the analysis of tumour heterogeneity at a much greater resolution than before, including and up to single-cell levels, potentially laying the groundwork for a standardized set of measures that can encompass overall tumour aneuploidy or CIN rates, in combination with accurate ITH measurement. Adding to the confusion, these three terms are often used almost interchangeably at times within the literature, with CIN particularly prone to being loosely defined. It has become increasingly clear that there is a need for consistent, clear use of terminology and an increased awareness of the close relationship between these three factors to accurately interpret the impact of aneuploidy, CIN and ITH in cancer resistance development.

We highlight here the importance of considering all of the potential causes and variables that might contribute to a given experimental or clinical dataset and of continuing to devise methods and lines of questioning that will allow the field to delve deeper into the cooperation between aneuploidy, CIN and ITH. We are not the first to attempt the disentanglement of two or more of the facets of CIN, aneuploidy and ITH, (Giam and Rancati 2015; Van Jaarsveld and Kops 2016; van den Bosch et al. 2022) but we hope this review re-iterates the importance of first deconstructing their potential mechanistic contributions, before we can begin to build a holistic overview of the many and complex processes involved in driving therapy resistance in cancer.

## Data Availability

Not applicable.
